# Drivers of COVID-19 vaccine uptake among rural populations in Madagascar: a cross-sectional study

**DOI:** 10.1186/s12889-024-20414-y

**Published:** 2024-10-17

**Authors:** Irina Kislaya, Diavolana Koecher Andrianarimanana, Valentina Marchese, Lalatiana Hosay, Rakotonavalona Rivomalala, Ramananjanahary Holinirina, Tahinamandranto Rasamoelina, Alexina Olivasoa Tsiky Zafinimampera, Sonya Ratefiarisoa, Olivette Totofotsy, Rivo Rakotomalala, Pia Rausche, Cheick Oumar Doumbia, Ariane Guth, Viola Pavoncello, Simon Veilleux, Zely Arivelo Randriamanantany, Jürgen May, Dewi Ismajani Puradiredja, Rivo Andry Rakotoarivelo, Daniela Fusco

**Affiliations:** 1 Department of Infectious Diseases Epidemiology, Bernhard Nocht Institute for Tropical Medicine, Bernhard-Nocht-Strasse 74, D-20359 Hamburg, Germany; 2 German Center for Infection Research (DZIF), Hamburg-Borstel-Lübeck-Riems, Germany; 3 Centre Hospitalier Universitaire (CHU) Androva, Mahajanga, Madagascar; 4 University of Mahajanga, Mahajanga, Madagascar; 5 Direction Régionale de la Santé Publique Boeny, Mahajanga, Madagascar; 6 Vaccination Program, Ministry of Public Health, Antanarivo, Madagascar; 7 Centre d’Infectiologie Charles Mérieux, Antananarivo, Madagascar; 8 University Clinical Research Center, University of Sciences, Techniques and Technologies of Bamako, Bamako, Mali; 9 Ministry of Public Health of Madagascar, Antananarivo, Madagascar; 10 University of Fianarantsoa, Fianarantsoa, Madagascar; 11 Centre Hospitalier Universitaire (CHU) Tambohobe, Fianarantsoa, Madagascar

**Keywords:** Public health, COVID-19, COVID-19 vaccination, Vaccination uptake, Vaccination willingness, Vaccination strategies

## Abstract

**Background:**

The WHO set the global immunisation threshold for COVID-19 at 70% to achieve worldwide protection against the disease. To date, global COVID-19 vaccine coverage is still below this threshold, in particular in several sub-Saharan African (SSA) countries, such as Madagascar. While factors influencing COVID-19 vaccine hesitancy have been widely explored in the past few years, research on drivers of COVID-19 vaccine uptake remains scarce. This study aimed at investigating drivers associated with COVID-19 vaccine uptake in the Boeny region of Madagascar.

**Methods:**

The study used a cross-sectional survey design to collect data on drivers of vaccine uptake from a sample of adults recruited from 12 healthcare facilities between November 2022 and February 2023. Relative and absolute frequencies were used to summarize participants’ characteristics. Prevalence ratios were estimated by Poisson regression to identify and compare sociodemographic and motivational drivers of vaccine uptake among those who were willing to get vaccinated against COVID-19 with those who had already been vaccinated.

**Results:**

A total of 928 participants aged between 18 and 76 years were included in the study. Among those recruited, 44.9% (*n* = 417) had already been vaccinated and 55.1% (*n* = 511) were willing to receive their first dose of COVID-19 vaccine on the day of the interview. The proportions of those respondents who live in urban areas (56.5% vs. 43.8%) and who have high school or university education (46.6% vs. 35.8%) were higher for the uptake group, whereas the proportion of employed respondents (66.3% vs. 56.5%) was higher among those willing to get vaccinated. Vaccine being free of charge (aPR = 1.77 [CI 95%: 1.45–2.17]) and being able to travel again (aPR = 1.61 [CI 95%: 1.30–1.98]) were the drivers most strongly associated with higher vaccine uptake after adjustment for sociodemographic factors.

**Conclusions:**

This study shows that actual COVID-19 vaccine uptake is influenced by a different set of factors than willingness to get vaccinated. Taking this difference in drivers into account can inform more tailored vaccination strategies to increase worldwide coverage.

## Background

Vaccination is a critical public health intervention for reducing disease burden and mortality. Despite its benefits, in 2019, the World Health Organization (WHO) declared vaccine hesitancy to be one of the top ten threats to global public health [1]. The COVID-19 pandemic has intensified the focus on vaccine hesitancy [2–7] with studies indicating variations in hesitancy due to factors like new information, or policies, or newly reported vaccine risks [2–5, 8, 9]. Vaccine hesitancy is defined as a “delay in acceptance or refusal of vaccination despite availability of vaccination services” [10], while vaccine willingness refers to the intent or motivation to be vaccinated [4]. Both concepts reflect the intentions that precede actual vaccines uptake or refusal [11].

Globally, COVID-19 vaccination uptake remains low, especially in many SSA countries [12].

Madagascar has the lowest COVID-19 vaccination rate worldwide. With only 9% of its population being vaccinated against COVID-19 with at least one dose by November 26, 2023, Madagascar falls considerably short in terms of the WHO’s target of 70% coverage [12, 13]. Low vaccination coverage in the country is primary due to infrastructural (shortage in healthcare personnel with adequate training on vaccine administration and pharmacological vigilance, shortage in cold chain equipment, information technology for and equipment for registry and control vaccine distribution), logistical (limited population access to healthcare facilities), and supply challenges [14]. In addition, the country joined the COVAX initiative comparatively late [15, 16] and had a neutral political stance towards COVID-19 vaccination. While vaccination was encouraged for at-risk groups, such as healthcare workers or older age groups (55 years old or more), it was not introduced as a mandatory requirement to resume social activities or travel [17]. Madagascar’s COVID-19 vaccination rollout begun in May 2021. Vaccination was implemented according to the national COVID-19 immunization plan [18], initially in the public primary care facilities where vaccines were available for the adult population free of charge. No further updates on vaccine recommendations for younger age groups were issued by the authorities. Various WHO-approved vaccine brands were used, namely Pfizer, Janssen, Covishield / AstraZeneca, Sinopharm, of which the Janssen vaccine was the most common one.

Several international efforts have aimed to boost coverage in the country, including the campaign that provided data for this study [19], the ‘*CO*VID-19 vaccination campaign in the *Bo*eny region of Madagascar: paving the road for worldwide vaccination coverage *go*al (CoBoGo)’. CoBoGo is a partnership between the Malagasy Ministry of Health, Malagasy academic institutions, and the Bernhard Nocht Institute for Tropical Medicine, launched under the financing umbrella of the Deutsche Gesellschaft für Internationale Zusammenarbeit GmbH aiming to deploy 25,000 doses of COVID-19 vaccines within six months, addressing infrastructural and logistical challenges in Boeny region.

An initial United Nations International Children’s Emergency Fund (UNICEF) survey in July 2021 indicated that about 53% of adults in Madagascar viewed COVID-19 vaccination as important and 29% intended to get vaccinated [18]. By September 2022, however, COVID-19 vaccine coverage was only 5.4%, revealing a significant behavioural gap between willingness and actual vaccine uptake that must be addressed to achieve coverage targets.

The WHO Technical Advisory Group on Behavioural Insights and Sciences for Health highlighted three major drivers of vaccine uptake: (1) an enabling environment, (2) social influences, and (3) motivations [20]. This framework has guided research to develop effective strategies to improve vaccination willingness and uptake. Conventional awareness campaigns seem to have had only a limited effect on vaccine uptake [11]. Extant research has shown that effective strategies to increase willingness to get vaccinated, include reducing barriers for sourcing and administration, fostering social and individual motivations, and building confidence in health workers [5, 8, 11, 20, 21]. However, far fewer studies have used actual vaccine uptake as their outcome comparing it to a willingness to get vaccinated [22], which is crucial to address the identified behavioural gap. As a part of the CoBoGo vaccination campaign, this study investigated the drivers of COVID-19 vaccine uptake in the Boeny region of Madagascar, employing the WHO framework previously described. We compared drivers among those willing to receive a vaccine with those already vaccinated, in order to strengthen the evidence base for more tailored and effective vaccination both, in Madagascar and similar contexts.

## Methods

### Study design and settings

This cross-sectional survey targeted adults (≥ 18 years old) in the Boeny region of the North-West Madagascar, conducted between November 2022 and February 2023, within a COVID-19 vaccination campaign implemented collaboratively by local authorities and academic partners.

### Study participants

The following eligibility criteria were applied to enroll study participants: (a) residency in the Boeny region; (b) age ≥ 18 years old; (c) having received at least one dose of a COVID-19 vaccine or willingness to get vaccinated on the interview day; and (d) ability to provide informed consent and participate in French or Malagasy.

### Sampling procedure and sample size determination

Study participants were recruited using a convenience sampling strategy across the 12 healthcare facilities that were included in the CoBoGo COVID-19 vaccination campaign in five Boeny municipalities, based on previously established collaborations between local authorities and academic partners.

At each site, survey staff conveniently approached and screened individuals visiting the healthcare facility for eligibility and recruited up to ten voluntary participants per day.

A target sample size of 948 individuals was determined using OpenEpi software to achieve 80% power to detect a 10% difference between groups, assuming 20% unexposed with the outcome, a significance level of 5%, and a design effect of 1.5 [23].

### Data collection

Data collection was conducted by twenty-four interviewers who completed a one-day training on all survey procedures and interviewing techniques, to ensure consistency and minimize interviewer and social desirability bias.

Face-to-face interviews were conducted in French or Malagasy, using a succinct paper-based questionnaire designed to minimize interference with the vaccination procedures. The questionnaire included sections on sociodemographic information, COVID-19 vaccination status, and willingness to get vaccinated, reasons for COVID-19 uptake, and sources of information on the vaccination campaign and took no longer than ten minutes to complete. The questionnaire was developed based on existing validated instruments used in previous studies [3] and was pre-tested to ensure linguistic and cultural appropriateness.

### Outcome variable

Self-reported vaccination status was assessed with the questions “Have you ever received a COVID-19 vaccine?”, and, if negative “Would you like to be vaccinated against COVID-19 today?”. Responses were combined into a dichotomous outcome “already vaccinated” vs. “willing to get vaccinated”.

### Independent variables

Independent variables included sociodemographic factors, such as age, residence type (rural/urban), education level (never attended school or incomplete primary school, primary or secondary school, high school or university education), employment status, and drivers for vaccine willingness and vaccine uptake. Drivers of vaccination were assessed on the basis of the three sub-categories (enabling environment, social influence and motivation) with multiple closed and open questions. Interviewers were instructed not to prompt the responses. Multiple answers were possible, for the purpose of the analysis, each category was considered as a dichotomous variable.

### Data management and analysis

Fieldwork supervisors performed daily data quality checks to identify and correct inconsistencies and data was entered into a Kobo toolbox database [24].

Analyses were conducted using the R version 4.3.1 [25] (base, car, ggplot2, sandwich, dplyr, Table 1). Relative and absolute frequencies summarized participants’ characteristics. The frequency of vaccination drivers and information sources was estimated for those vaccinated and those willing to get vaccinated. Crude and adjusted prevalence ratios were estimated using Poisson regression with robust standard errors, as they provide a more direct and interpretable measure of association in cross-sectional studies compared to odds ratios, especially when the outcome is not rare [26]. Informed by WHO’s considerations on COVID-19 vaccination acceptance and uptake [20], we included in the regression model three categories of drivers of vaccine uptake: (a) enabling environment (vaccine being free of charge, urban residency, occupation), (b) social influences (being encouraged by others to get vaccinated), (c) motivational factors (protection of own health, protection of family and friends’ health, protection of the community, return to travel, to social life, to work, education and school activities), and (d) socio-demographic factors (age group, education). Observations with missing values were excluded from the analysis. The significance level was set at 5%.

### Ethical considerations

This study complied with legal and ethical requirements, receiving approval from the Ethics Committee Hamburg State Medical Chamber (protocol number: 2021-10550-BO-ff) and the National Ethics Committee of Madagascar (CERBM: IORGO000851 N°81 MSANP/SG/AMM/CERBM). Survey participation was voluntary, with no monetary incentives. Written informed consent was obtained from all participants, and an impartial witness was involved for illiterate participants. Participants willing to get vaccinated were accompanied to the vaccination service immediately after the interview to get a COVID-19 vaccine. Participants were assured of data privacy and encouraged to answer questions honestly. To ensure confidentiality, all collected data were pseudonomysed and stored securely on a password-protected Kobo toolbox database accessible only to authorized research personnel. Physical questionnaires were kept in locked cabinets to prevent unauthorized access. The study posed minimal risk to participants, primarily involving the time taken for interviews. Participants were informed that their participation would contribute to improving public health strategies for vaccine uptake in the region.

## Results

### Participants characteristics

Overall 928 individuals were included in this study. The majority of participants were aged 18–29 years (43.6%), with a nearly equal split between rural and urban residents (50.2% vs. 49.8%, respectively; Table 1).

61.9% (*n* = 569) of participants were employed, and 40.6% (*n* = 377) had a secondary school or university education.

Among recruited, 44.9% (*n* = 417) were already vaccinated, while 55.1% (*n* = 511) were willing to receive their first dose of the COVID-19 vaccine. Urban residency (56.5%, *n* = 238 vs. 43.8%, *n* = 224) and high school or university education (46.6%, *n* = 194 vs. 35.8%, *n* = 183) were more common in the vaccinated group, whereas employment was more common in the willing group (66.3%, *n* = 335 vs. 56.5%, *n* = 234).

**Table 1:** Characteristics of study participants, Boeny, Madagascar, November 2022-February 2023

	Total sample	Willingness	Uptake
	*n* = 928	*n* = 511	*n* = 417
**Age groups**			
18–29	405 (43.6%)	225 (44.0%)	180 (43.2%)
30–39	227 (24.5%)	129 (25.2%)	98 (23.5%)
40+	296 (31.9%)	157 (30.7%)	139 (33.3%)
**Residence type**			
Rural area	466 (50.2%)	287 (56.2%)	179 (42.9%)
Urban area	462 (49.8%)	224 (43.8%)	238 (57.1%)
**Occupation**			
Employed	569 (61.9%)	335 (66.3%)	234 (56.5%)
Not employed	350 (38.1%)	170 (33.7%)	180 (43.5%)
**Education level**			
Incomplete/No school education	249 (26.8%)	134 (26.2%)	115 (27.6%)
Primary/middle school	301 (32.4%)	194 (38.0%)	107 (25.7%)
High school/University	377 (40.6%)	183 (35.8%)	194 (46.6%)

### Sources of information about COVID-19 vaccination

**Fig. 1:**
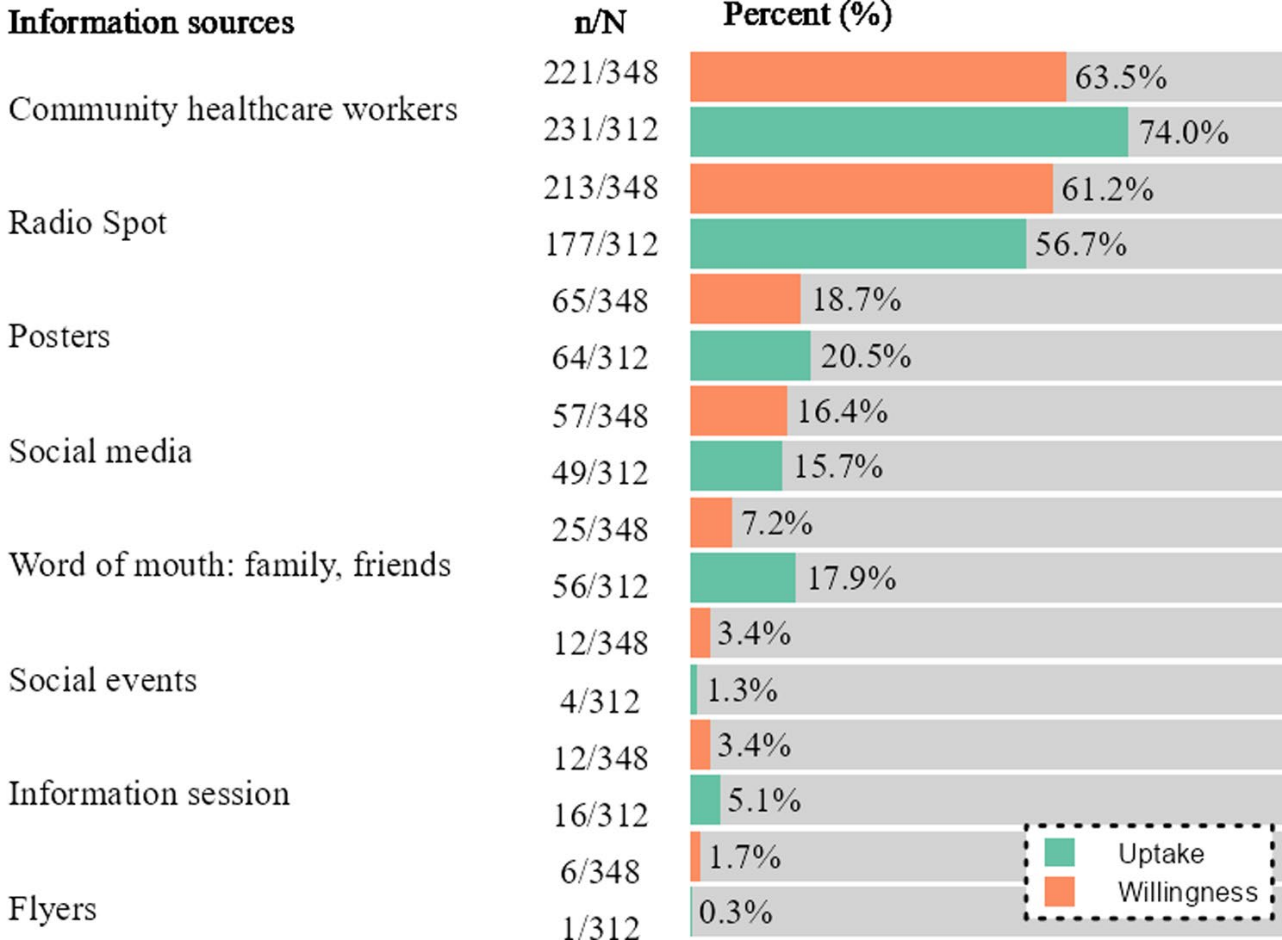
Information sources on vaccination campaign in Boeny, Madagascar, November 2022-February 2023 Legend Information sources were assessed among participants aware of the CoBoGo campaign (vaccinated *n* = 312 and willing *n* = 348)

Survey participants were asked for their main sources of information about COVID-19 vaccination, specifically concerning the ongoing CoBoGo campaign. Most were aware of the CoBoGo campaign (74.8%, *n* = 312 vaccinated and 68.1%, *n* = 348 willing). Participants aware of the CoBoGo campaign reported community healthcare workers (CHW) (74.0%, *n* = 231 vaccinated and 63.5%, *n* = 221 willing), followed by radio (56.7%, *n* = 177 vaccinated and 61.2%, *n* = 213 willing), and posters (20.5%, *n* = 64 vaccinated and 18.7%, *n* = 65 willing), as the top information sources (Fig. 1). Family and friends (17.9%, *n* = 56) and social media (e.g. Facebook) (16.4%, *n* = 57) were also notable sources for vaccinated and willing participants, respectively.

### Drivers of vaccination willingness and vaccine uptake

**Fig. 2:**
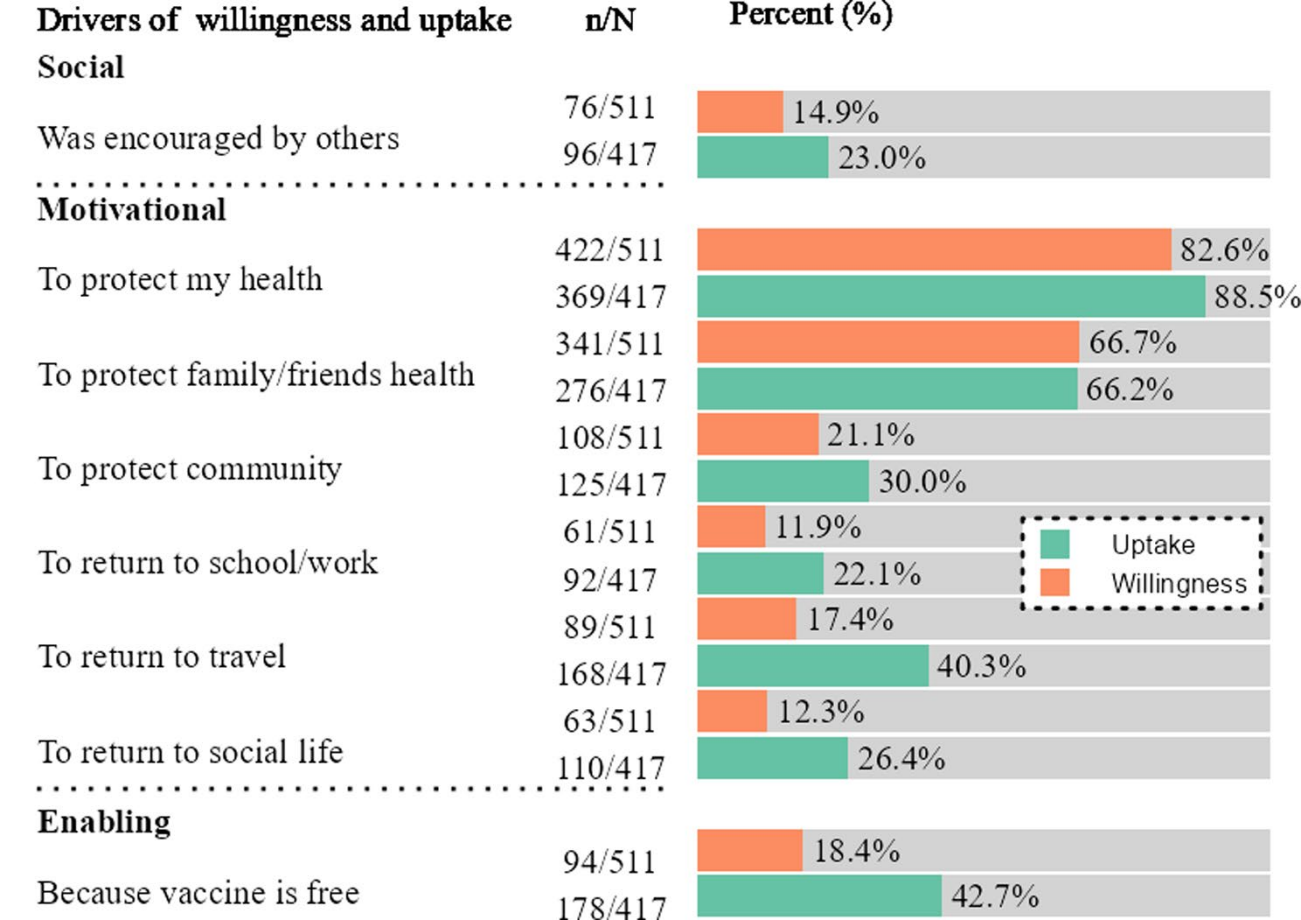
Drivers of COVID-19 vaccination uptake and willingness reported by those already vaccinated and those willing to get vaccinated against COVID-19 in Boeny, Madagascar, November 2022-February 2023

The primary drivers for vaccination among the vaccinated were “To protect my health” (88.5%, *n* = 369), “To protect my family/friends’ health” (66.2%, *n* = 276), and “Because the vaccine is free” (42.7%, *n* = 178) and “To return to travel” (40.3%, *n* = 168) (Fig. 2).

For those willing to get vaccinated the main drivers were the protection of their own personal health (82.6%, *n* = 422), followed by protecting family and friends’ health (66.7%, *n* = 341), and protection of the community health (21.1%, *n* = 108) (Fig. 2). Other factors, such as “Because the vaccine is free” (18.4%, *n* = 94) and “To return to travel” (17.4%, *n* = 89) were less frequently mentioned.

Being encouraged by others, returning to work/school, and resuming a social life were among the least frequently reported drivers of COVID-19 vaccination in both groups. Those participants who stated that they had been encouraged by others specified that they had most frequently receiving the encouragement from healthcare workers and the Ministry of Health. In contract, having received encouragement from community leaders as encouraging vaccination was only mentioned once.

### Factors associated with vaccine uptake

Table 2 reports the adjusted prevalence ratios (aPR) with 95% confidence intervals (CI 95%) of COVID-19 vaccine uptake as compared with willingness. Significant positive associations with vaccine uptake included free vaccination availability (aPR = 1.77, CI 95%:1.45–2.17), and the prospect of resuming travelling (aPR = 1.61, CI 95%:1.30–1.98). Urban residency was associated with a 31% higher vaccine uptake (aPR = 1.31, 95% CI: 1.07–1.60), suggesting that accessibility of healthcare facilities in urban areas may play a critical role in vaccine uptake.

Conversely, the encouragement by others (aPR = 0.65, CI 95%:0.48–0.88) and protecting family and friends’ health (aPR = 0.81, CI 95%:0.67–0.98) showed significant associations with reduced uptake as compared with willingness.

**Table 2:** Crude (PR) and adjusted prevalence ratios (aPR) COVID-19 vaccine uptake vs. willingness to get vaccinated in Boeny, Madagascar, November 2022-February 2023

	*N*	*n*	*p*	PR	aPR
**Age group**					
18-29 (ref)	405	180	44.4	1	1
30-39	227	98	43.2	0.97 (0.76; 1.24)	1.07 (0.88; 1.30)
40+	296	139	47.0	1.06 (0.85; 1.32)	1.17 (0.97; 1.40)
**Urbanization**					
Rural (ref)	466	179	38.4	1	1
Urban	462	238	51.5	1.34 (1.16; 1.55)	1.31 (1.07; 1.60)
**Occupation**					
Employed (ref)	569	234	41.1	1	1
Not employed	350	234	51.4	1.25 (1.08; 1.44)	1.19 (0.99; 1.41)
**Education**					
Incomplete/No formal education	249	115	46.2	1	1
Primary/Middle	301	107	35.5	0.77 (0.63; 0.94)	0.87 (0.71; 1.06)
High school/University	377	194	51.5	1.11 (0.94; 1.32)	1.11 (0.91; 1.35)
**To protect my health**					
No (ref)	137	48	35.0	1	1
Yes	791	369	46.6	1.33 (1.06; 1.66)	1.22 (0.95; 1.56)
**To protect family/friends´ health**					
No (ref)	311	141	45.3	1	1
Yes	617	276	44.7	0.99 (0.85; 1.15)	0.81 (0.67; 0.98)
**To protect community**					
No (ref)	695	292	42.0	1	1
Yes	233	125	53.6	1.28 (1.09; 1.49)	0.78 (0.60; 1.03)
**To return to school/work**					
No (ref)	775	325	41.9	1	1
Yes	153	92	60.1	1.43 (1.21; 1.7)	1.10 (0.88; 1.36)
**To return to travel**					
No (ref)	671	249	37.1	1	1
Yes	257	168	65.4	1.76 (1.52; 2.04)	1.61 (1.30; 1.98)
**To return to social life**					
No (ref)	755	307	40.7	1	1
Yes	173	110	63.6	1.56 (1.33; 1.84)	1.05 (0.74; 1.46)
**Was encouraged by others**					
No (ref)	756	321	42.5	1	1
Yes	172	96	55.8	1.31 (1.11; 1.56)	0.65 (0.48; 0.88)
**Because the vaccine is free**					
No (ref)	656	239	36.4	1	1
Yes	272	178	65.4	1.80 (1.56; 2.07)	1.77 (1.45; 2.17)

Note N – total number of observations, n -number of vaccinated, p – proportion of vaccine uptake,

PR – crude prevalence ratio, aPR - adjusted prevalence ratio

## Discussion

This study aimed to identify drivers of COVID-19 vaccine uptake as compared with willingness within the context of the CoBoGo vaccination campaign in Madagascar. Our findings show that key drivers of high vaccine uptake includ the vaccine being free of charge (aPR = 1.77 [CI 95%: 1.45–2.17]), and the prospect of returning to travel (aPR = 1.61 [CI 95%: 1.30–1.98]), followed by urban residency (aPR = 1.31, 95% CI: 1.07–1.60).

Free of charge vaccination has been shown to reduce inequalities and to facilitate uptake in high-income countries among vulnerable populations with suboptimal vaccination coverage [27, 28]. It is also crucial in low-income settings, where over 80% of the population lives in extreme poverty [29]. In fact, having access to free vaccines has been recognized by the populations as a desirable feature of vaccination campaigns in 34 African countries [30]. Thus, ensuring an enabling environment in Madagascar and other limited-resource settings within which vaccines are offered free of charge can enhance and sustain vaccine uptake in adult populations.

The prospect of taking up travel again was a significant motivational driver of vaccine uptake, reported by 40.5% of vaccinated participants. While domestic travel did not require vaccination [17], international travel did. This may have been of particular relevance for those with higher levels of education within the sample, who tend to have more interest and capacity for international travel. Other studies corroborate that resuming international travel plays a critical role in influencing vaccine acceptance and uptake in both low- and high- income countries [4, 6].

Encouragement by others (aPR = 0.65 [CI 95%: 0.48–0.88]) was a less relevant driver for those already vaccinated, suggesting that the decision to get vaccinated may be more autonomous for this group. While some studies have shown that encouragement from trusted individuals, such as relatives, friends, and healthcare workers, can influence vaccine willingness for COVID-19 and other diseases [31, 32], the final decision to get vaccinated may be more autonomous, and internal [33]. This complexity of the decision-making process suggests a need for further longitudinal studies to better understand these dynamics and inform communication strategies that leverage social encouragement effectively to increase vaccine uptake [34].

In addition, this study shows that personal motivations, particularly self-protection (88.5% uptake, 82.6% willingness), were primary drivers for both groups. Family and friends’ health was also important, while community health was less frequently reported (21.2-30.0%). This finding contrasts with the widely held assumption that communities in low- and middle-income (LMICs), give greater importance to collective well-being [35] than the more individualistic high-income societies [36, 37]. These insights are crucial for designing communication strategies around the benefits of vaccination.

Consistent with other studies in LMICs and SSA [22], urban residency was associated with higher COVID-19 vaccine uptake due to better access to services [38, 39]. This is particularly concerning for Madagascar where most of the population lives in remote areas with limited access to healthcare [40]. Our data highlight the need for vaccination strategies that put greater emphasis on reaching remote populations. Community health workers (CHWs) can play an important role in helping to create an enabling environment for vaccination, as they are trusted sources of information and are effective in reaching isolated and marginalised populations [41–43]. In countries, such as Madagascar, where the health system strongly relies on CHWs [44], implementing supportive measures for CHWs, such as training and access to health records, can optimize vaccine coverage [45, 46].

Finally, in our study setting, social media are less frequently used to gather information about COVID-19 vaccinations than, for example, more traditional media, such as radio. This element is particularly relevant when considering the use of social media for the implementation of health-related awareness and information programs for the general population. In Madagascar, the use of social media for vaccination-related awareness raising campaign might not be yet as effective as in other countries [47].

This study is among the few in SSA comparing COVID-19 vaccine willingness and uptake and the first to explore social attitudes towards vaccination in Madagascar. The findings contribute to the global knowledge about drivers of vaccine uptake and provide valuable insights for adapting public health strategies in Madagascar and other countries sharing similar challenges and cultural contexts. However, this study is not without limitations. The cross-sectional survey design limits causal conclusions, the convenience sampling strategy can introduce some degree of selection bias, potentially favoring participants with higher education and better access to healthcare. This may limit the generalizability of our findings to the wider population. On the other hand, the convenience sampling strategy allowed us to optimize fieldwork time and costs in the context of externally low COVID-19 vaccine coverage in the region. Additionally, to collect the data in healthcare facilities with as little disruption as possible for vaccination rollout, we designed a succinct survey instrument, which did not address vaccine characteristics (safety, efficacy, vaccine brand), information on participants’ sex was also not collected, potentially leading to unmeasured confounding bias. Moreover, the use of self-reported data could lead to some social desirability bias, widely acknowledged in research on vaccine willingness [3, 22, 30]. Our methodological approach to the data collection helped to minimize this type of bias through non-judgemental interviewing techniques and question formulation.

## Conclusions

In summary, this study demonstrates that factors influencing COVID-19 vaccine uptake differ from those influencing willingness to get vaccinated. The most influential drivers associated with higher vaccination uptake alongside access to health services included the vaccine being free of charge and the desire to resume travel, indicating that both economic and mobility incentives played a crucial role in vaccination decisions in Malagasy population.

Understanding and addressing these drivers along the continuum of decision-making around vaccinations can help to improve vaccination strategies beyond the COVID-19 pandemic and develop tailored communication campaingns in Madagascar and other countries sharing similar challenges.

## Data Availability

Raw data used in the current study are available from the corresponding author on reasonable request and will be freely available to researchers who wish to use them for non-commercial purposes.

## Abbreviations

aPR: Adjusted prevalence ratio
CI: Confidence interval
CERBM: National Biomedical Research Ethics Committee in Madagascar
CHW: Community healthcare worker
CoBoGo: COVID-19 vaccination campaign in the Boeny region of Madagascar: paving the road for Worldwide vaccination coverage goal
COVID-19: COronaVIrus Disease 19
LMIC: Low- and middle-income countries
SSA: Sub-Saharan African
UNICEF: United Nations Children’s Fund
WHO: World Health Organization

## References

[1] Ten health issues WHO will. tackle this year [Internet]. [cited 2023 Jul 24]. https://www.who.int/news-room/spotlight/ten-threats-to-global-health-in-2019

[2] Solís Arce JS, Warren SS, Meriggi NF, Scacco A, McMurry N, Voors M, et al. COVID-19 vaccine acceptance and hesitancy in low- and middle-income countries. Nat Med. 2021;27(8):1385–94. 34272499 10.1038/s41591-021-01454-y PMC8363502

[3] Faye SLB, Krumkamp R, Doumbia S, Tounkara M, Strauss R, Ouedraogo HG, et al. Factors influencing hesitancy towards adult and child COVID-19 vaccines in rural and urban West Africa: a cross-sectional study. BMJ Open. 2022;12(4):e059138. 35418436 10.1136/bmjopen-2021-059138 PMC9013792

[4] Lazarus JV, Wyka K, White TM, Picchio CA, Gostin LO, Larson HJ, et al. A survey of COVID-19 vaccine acceptance across 23 countries in 2022. Nat Med. 2023;29(2):366–75. 36624316 10.1038/s41591-022-02185-4

[5] Kalu ME, Oyinlola O, Ibekaku MC, Adandom II, Iwuagwu AO, Ezulike CJ, et al. A mapping review on the Uptake of the COVID-19 vaccine among adults in Africa using the 5As Vaccine Taxonomy. Am J Trop Med Hyg. 2022;106(6):1688–97. 35533697 10.4269/ajtmh.21-0515 PMC9209920

[6] Lazarus JV, Wyka K, White TM, Picchio CA, Rabin K, Ratzan SC, et al. Revisiting COVID-19 vaccine hesitancy around the world using data from 23 countries in 2021. Nat Commun. 2022;13:3801. 35778396 10.1038/s41467-022-31441-x PMC9247969

[7] Abubakari SW, Workneh F, Asante KP, Hemler EC, Madzorera I, Wang D, et al. Determinants of COVID-19 vaccine readiness and hesitancy among adults in sub-saharan Africa. PLOS Glob Public Health. 2023;3(7):e0000713. 37450441 10.1371/journal.pgph.0000713 PMC10348558

[8] Roy DN, Biswas M, Islam E, Azam MS. Potential factors influencing COVID-19 vaccine acceptance and hesitancy: a systematic review. PLoS ONE. 2022;17(3):e0265496. 35320309 10.1371/journal.pone.0265496 PMC8942251

[9] Deml MJ, Githaiga JN. Determinants of COVID-19 vaccine hesitancy and uptake in sub-saharan Africa: a scoping review. BMJ Open. 2022;12(11):e066615. 36400736 10.1136/bmjopen-2022-066615 PMC9676416

[10] MacDonald NE. Vaccine hesitancy: definition, scope and determinants. Vaccine. 2015;33(34):4161–4. 25896383 10.1016/j.vaccine.2015.04.036

[11] Brewer NT. What Works to Increase Vaccination Uptake. Acad Pediatr. 2021;21(4, Supplement):S9–16. 10.1016/j.acap.2021.01.017 33958099

[12] WHO Coronavirus (COVID-19.) Dashboard [Internet]. [cited 2023 Jul 24]. https://covid19.who.int

[13] Achieving 70%. COVID-19 Immunization Coverage by Mid-2022 [Internet]. [cited 2023 Jul 24]. https://www.who.int/news/item/23-12-2021-achieving-70-covid-19-immunization-coverage-by-mid-2022

[14] Decouttere C, De Boeck K, Vandaele N. Advancing sustainable development goals through immunization: a literature review. Glob Health. 2021;17(1):95. 10.1186/s12992-021-00745-w PMC8390056 34446050

[15] Rasambainarivo F, Ramiadantsoa T, Raherinandrasana A, Randrianarisoa S, Rice BL, Evans MV, et al. Prioritizing COVID-19 vaccination efforts and dose allocation within Madagascar. BMC Public Health. 2022;22(1):724. 35413894 10.1186/s12889-022-13150-8 PMC9002044

[16] COVAX [Internet]. [cited 2023 Jul 24]. https://www.who.int/initiatives/act-accelerator/covax

[17] Plateforme Vaksiny pour la gestion des vaccins à Madagascar [Internet]. Unité de Gouvernance Digitale. 2021 [cited 2023 Jul 25]. https://digital.gov.mg/2021/09/08/plateforme-vaksiny-pour-la-gestion-des-vaccins-a-madagascar/

[18] Ministre de la Santé Publique de Madagascar. Plan National de Déploiement et de Vaccination (PNDV) contre la Covid-19 à Madagascar. 2021.

[19] Amt A. German Federal Foreign Office. [cited 2023 Jul 25]. COVID-19: Germany’s commitment to fair distribution of vaccines. https://www.auswaertiges-amt.de/en/aussenpolitik/themen/covax/2396914

[20] WHO technical advisory group on behavioural insights and sciences for health. Behavioural considerations for acceptance and uptake of COVID-19 vaccines [Internet]. 2020 [cited 2023 Jul 24]. https://www.who.int/publications-detail-redirect/9789240016927

[21] Brewer NT, Chapman GB, Rothman AJ, Leask J, Kempe A. Increasing vaccination: putting Psychological Science Into Action. Psychol Sci Public Interest. 2017;18(3):149–207. 29611455 10.1177/1529100618760521

[22] Whitehead HS, Songo J, Phiri K, Kalande P, Lungu E, Phiri S, et al. Correlates of uptake of COVID-19 vaccines and motivation to vaccinate among Malawian adults. Hum Vaccines Immunother. 2023;19(2):2228168. 10.1080/21645515.2023.2228168 PMC10332229 37394430

[23] Fleiss JL. Statistical methods for Rates and proportions. 3rd ed. John Wiley & Sons, Inc; 2003.

[24] KoboToolbox [Internet]. [cited 2023 Jul 24]. KoboToolbox. https://www.kobotoolbox.org/

[25] R: The R Project for Statistical Computing [Internet]. [cited 2023 Jul 24]. https://www.r-project.org/

[26] Tamhane AR, Westfall AO, Burkholder GA, Cutter GR. Prevalence odds ratio versus prevalence ratio: choice comes with consequences. Stat Med. 2016;35(30):5730–5. 27460748 10.1002/sim.7059 PMC5135596

[27] Crawshaw AF, Farah Y, Deal A, Rustage K, Hayward SE, Carter J, et al. Defining the determinants of vaccine uptake and undervaccination in migrant populations in Europe to improve routine and COVID-19 vaccine uptake: a systematic review. Lancet Infect Dis. 2022;22(9):e254–66. 35429463 10.1016/S1473-3099(22)00066-4 PMC9007555

[28] Eiden AL, Barratt J, Nyaku MK. A review of factors influencing vaccination policies and programs for older adults globally. Hum Vaccines Immunother. 2023;19(1):2157164. 10.1080/21645515.2022.2157164 PMC9980618 36656057

[29] World Bank Open. Data [Internet]. [cited 2023 Jul 25]. World Bank Open Data. https://data.worldbank.org

[30] Anjorin AA, Odetokun IA, Abioye AI, Elnadi H, Umoren MV, Damaris BF, et al. Will africans take COVID-19 vaccination? PLoS ONE. 2021;16(12):e0260575. 34851998 10.1371/journal.pone.0260575 PMC8635331

[31] Kalunga L, Bulut E, Chen Z, Li Y, Ivanek R. Increasing vaccine uptake among employees within the non-health related critical infrastructure sectors: a review. Hum Vaccines Immunother. 2023;19(1):2135852. 10.1080/21645515.2022.2135852 PMC9980543 36628470

[32] Froes F, Morais A, Hespanhol V, Nogueira R, Carlos JS, Jacinto N, et al. The Vacinómetro^®^ initiative: an eleven-year monitorization of influenza vaccination coverage rates among risk groups in Portugal. Pulmonology. 2022;28(6):427–30. 35501279 10.1016/j.pulmoe.2022.03.005

[33] Schmitz M, Luminet O, Klein O, Morbée S, Van den Bergh O, Van Oost P, et al. Predicting vaccine uptake during COVID-19 crisis: a motivational approach. Vaccine. 2022;40(2):288–97. 34961635 10.1016/j.vaccine.2021.11.068 PMC8626229

[34] Tjilos M, Tamlyn AL, Ragan EJ, Assoumou SA, Barnett KG, Martin P, et al. Community members have more impact on their neighbors than celebrities: leveraging community partnerships to build COVID-19 vaccine confidence. BMC Public Health. 2023;23(1):350. 36797724 10.1186/s12889-023-15198-6 PMC9933023

[35] Pelham B, Hardin C, Murray D, Shimizu M, Vandello J. A truly global, non-WEIRD examination of collectivism: The Global Collectivism Index (GCI). Curr Res Ecol Soc Psychol. 2022;3:100030.

[36] Nikolaev B, Boudreaux C, Salahodjaev R. Are individualistic societies less equal? Evidence from the parasite stress theory of values. J Econ Behav Organ. 2017;138:30–49.

[37] Gorodnichenko Y, Roland G. Individualism, innovation, and long-run growth. Proc Natl Acad Sci. 2011;108(supplement4):21316–9. 22198759 10.1073/pnas.1101933108 PMC3271573

[38] Tessema ZT, Worku MG, Tesema GA, Alamneh TS, Teshale AB, Yeshaw Y, et al. Determinants of accessing healthcare in Sub-saharan Africa: a mixed-effect analysis of recent demographic and health surveys from 36 countries. BMJ Open. 2022;12(1):e054397. 35105635 10.1136/bmjopen-2021-054397 PMC8804632

[39] Bayati M, Noroozi R, Ghanbari-Jahromi M, Jalali FS. Inequality in the distribution of Covid-19 vaccine: a systematic review. Int J Equity Health. 2022;21(1):122. 36042485 10.1186/s12939-022-01729-x PMC9425802

[40] Weiss DJ, Nelson A, Gibson HS, Temperley W, Peedell S, Lieber A, et al. A global map of travel time to cities to assess inequalities in accessibility in 2015. Nature. 2018;553(7688):333–6. 29320477 10.1038/nature25181

[41] Vaughan K, Kok MC, Witter S, Dieleman M. Costs and cost-effectiveness of community health workers: evidence from a literature review. Hum Resour Health. 2015;13(1):71. 26329455 10.1186/s12960-015-0070-y PMC4557864

[42] Ryabov I. Cost-effectiveness of Community Health Workers in controlling diabetes epidemic on the U.S.–Mexico border. Public Health. 2014;128(7):636–42. 24999158 10.1016/j.puhe.2014.05.002

[43] The cost-effectiveness. of community health workers delivering free diarrhoea treatment: evidence from Uganda - PMC [Internet]. [cited 2023 Jul 26]. https://www.ncbi.nlm.nih.gov/pmc/articles/PMC8757489/10.1093/heapol/czab120 PMC8757489 34698342

[44] Evans MV, Andréambeloson T, Randriamihaja M, Ihantamalala F, Cordier L, Cowley G, et al. Geographic barriers to care persist at the community healthcare level: evidence from rural Madagascar. PLOS Glob Public Health. 2022;2(12):e0001028. 36962826 10.1371/journal.pgph.0001028 PMC10022327

[45] World Health Organization. WHO guideline on health policy and system support to optimize community health worker programmes. 2018. 30431747

[46] Naimoli JF, Perry HB, Townsend JW, Frymus DE, McCaffery JA. Strategic partnering to improve community health worker programming and performance: features of a community-health system integrated approach. Hum Resour Health. 2015;13(1):46. 26323276 10.1186/s12960-015-0041-3 PMC4556219

[47] Huo J, Desai R, Hong YR, Turner K, Mainous AG, Bian J. Use of Social Media in Health Communication: findings from the Health Information National Trends Survey 2013, 2014, and 2017. Cancer Control J Moffitt Cancer Cent. 2019;26(1):1073274819841442. 10.1177/1073274819841442 PMC6475857 30995864

